# P-260. Factors Associated with HIV Viral Suppression among People with ART, Kyrgyz Republic 2021-2023

**DOI:** 10.1093/ofid/ofaf695.481

**Published:** 2026-01-11

**Authors:** Akylai Kubatova, Nassyat Kemelbek, Roberta Horth, Dilyara Nabirova

**Affiliations:** Central Asia Advanced Field Epidemiology Training Program, Chuy, Kyrgyzstan; Central Asia FETP, Bishkek, Bishkek, Kyrgyzstan; US Centers for Disease Control and Prevention, Dulles, Virginia; CDC Central Asia office, Almaty, Almaty, Kazakhstan

## Abstract

**Background:**

Sustained viral suppression is a critical measure of the effectiveness of antiretroviral therapy (ART) and a vital component in achieving the UNAIDS "95-95-95" strategy, which seeks to ensure that 95% of people with HIV on ART attain viral load suppression by 2030. In the Kyrgyz Republic, where HIV incidence continues to rise, an increasing number of people are on ART. Understanding the factors associated with viral suppression is important for interrupting HIV transmission.

**Table 1.**
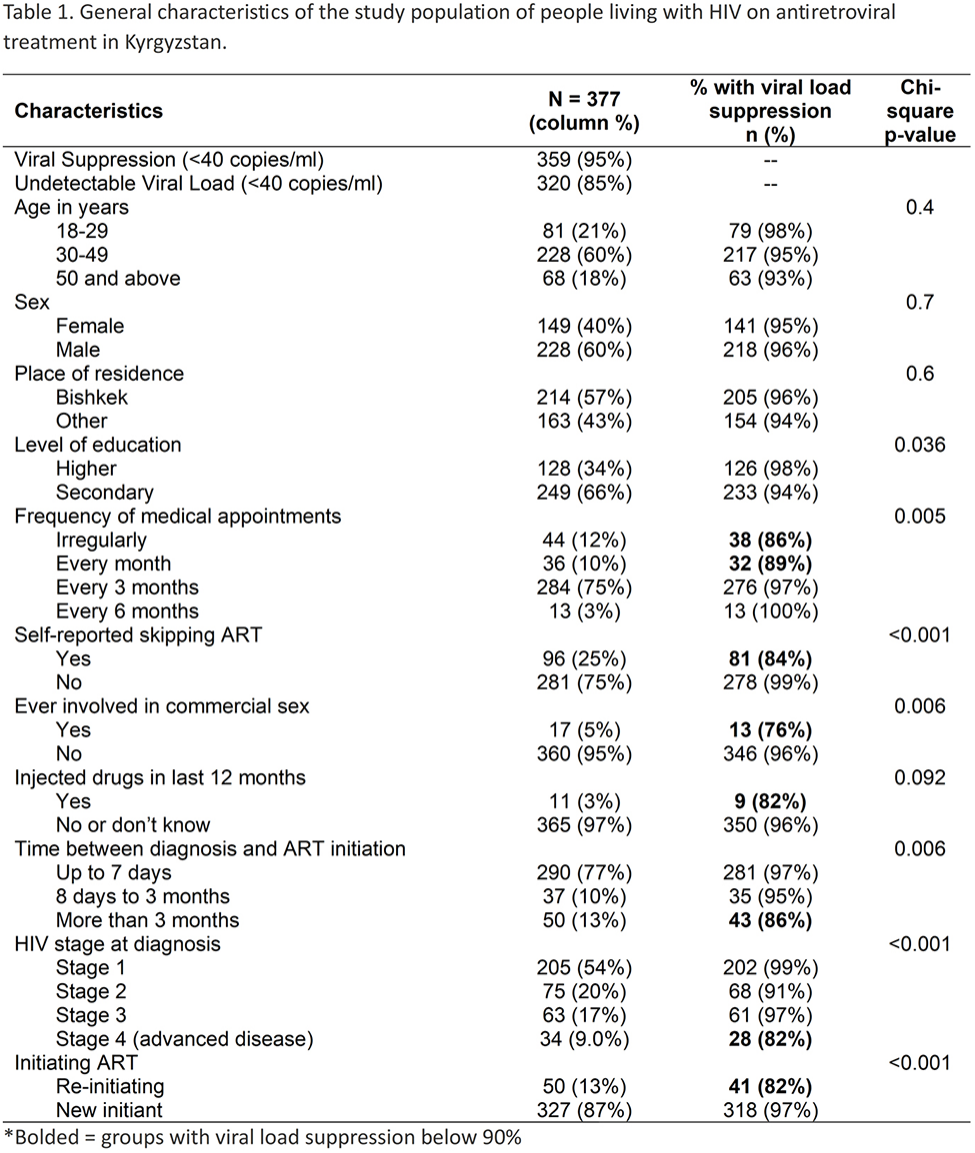
General characteristics of the study population of people living with HIV on antiretroviral treatment in Kyrgyzstan, 2023.

**Table 2.**
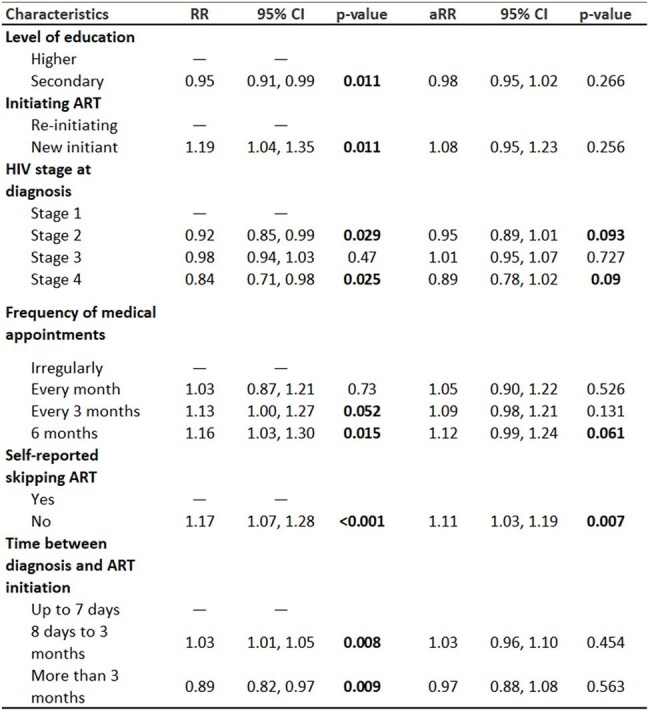
Factors associated with viral load suppression among people living with HIV on antiretroviral treatment in Kyrgyzstan, 2023 RR: relative risk from bivariable Poisson regression estimators. aRR: adjusted relative risk from multivariable Poisson regression including all variables listed in the table

**Methods:**

We conducted a retrospective cohort study among adults >18 years old with HIV in Kyrgyzstan. Participants included people initiating or reinitiating ART from June 2021 to June 2023 and who were on ART for at least 6 months. We performed logistic regression to identify factors associated with viral load suppression, defined as >200 copies/ml based on the most recent viral load test.

**Results:**

Among 377 participants, 95% achieved HIV viral suppression and 85% had undetectable viral load (≤40 copies/ml) (Table 1). Participants were mostly male (60%), 30-49 years old (60%), and self-employed (49%). All were on a dolutegravir containing ART. The proportion of those with viral suppression was below 90% for people who were re-initiating ART, with advanced HIV disease at diagnosis, who started ART more than 3 months after their HIV diagnosis, who injected drugs in last 12 months, and with monthly or unclear frequency of ART appointments. One-quarter of people self-reported an interruption in ART. People without skipped ART doses had higher likelihood of being virally suppressed (adjusted relative risk: 1.11 (95% confidence interval: 1.03-1.19) (Table 2).

**Conclusion:**

The results of the study show a high level of viral suppression and Kyrgyzstan is achieving the target of 95% viral load suppression among PLHIV, even though one-quarter of participants reported skipping ART doses. Understanding the frequency and reasons for missed doses is important to maintain high viral suppression.

**Disclosures:**

All Authors: No reported disclosures

